# Spatial Distribution of Underweight, Overweight and Obesity among Women and Children: Results from the 2011 Uganda Demographic and Health Survey

**DOI:** 10.3390/ijerph10104967

**Published:** 2013-10-11

**Authors:** Kedir N. Turi, Mary J. Christoph, Diana S. Grigsby-Toussaint

**Affiliations:** 1 Department of Kinesiology and Community Health, University of Illinois at Urbana-Champaign, 1206 S. Fourth Street, 80B Huff Hall, Champaign, IL 61820, USA; E-Mails: knturi@illinois.edu (K.N.T.); mchrstp2@illinois.edu (M.J.C.); 2 Division of Nutritional Sciences, University of Illinois at Urbana-Champaign, 1206 S. Fourth Street, 2019 Huff Hall, Champaign, IL 61820, USA

**Keywords:** obesity, Uganda, sub-Saharan Africa, spatial epidemiology, geographic information systems

## Abstract

While undernutrition and infectious diseases are still persistent in developing countries, overweight, obesity, and associated comorbidities have become more prevalent. Uganda, a developing sub-Saharan African country, is currently experiencing the public health paradox of undernutrition and overnutrition. We utilized the 2011 Uganda Demographic and Health Survey (DHS) to examine risk factors and hot spots for underweight, overweight, and obesity among adult females (*N* = 2,420) and their children (*N* = 1,099) using ordinary least squares and multinomial logit regression and the ArcGIS Getis-Ord Gi* statistic. Overweight and obese women were significantly more likely to have overweight children, and overweight was correlated with being in the highest wealth class (OR = 2.94, 95% CI = 1.99–4.35), and residing in an urban (OR = 1.76, 95% CI = 1.34–2.29) but not a conflict prone (OR = 0.48, 95% CI = 0.29–0.78) area. Underweight clustered significantly in the Northern and Northeastern regions, while overweight females and children clustered in the Southeast. We demonstrate that the DHS can be used to assess geographic clustering and burden of disease, thereby allowing for targeted programs and policies. Further, we pinpoint specific regions and population groups in Uganda for targeted preventive measures and treatment to reduce the burden of overweight and chronic diseases in Uganda.

## 1. Introduction

As many developing countries undergo economic, epidemiologic, and nutrition transitions, the coexistence of undernutrition and overnutrition has emerged as a public health paradox [1]. While undernutrition and infectious diseases persist, increased levels of overweight, obesity, and associated comorbidities have accompanied the nutrition transition in developing nations, and now cause a significant burden of disease [2]. Increased rates of overweight and obesity, as well as associated chronic diseases such as hypertension and diabetes, have been recently observed in many developing countries [2,3,4]. Noncommunicable diseases account for over 60% of all global deaths [5], with 80% now occurring in low- or middle-income countries [6]. One region undergoing rapid and sweeping changes in terms of demography, nutrition, and health is sub-Saharan Africa [7,8]. Overweight and obesity in the region increased 35% between 1992 and 2005 [8], and at least one study has shown that hypertension quadrupled from 2005 to 2008 to an overall prevalence of 16.2% [9]. Additionally, Hall *et al.* showed that diabetes prevalence varied from 1% to 12% of the population for parts of the region, costing an estimated $8,836 per patient or a total of $67.03 billion per year [10].

Understanding the geographic distribution of obesity is integral to designing interventions that target high-risk areas. Although spatial clustering and other geographic information systems (GIS) techniques are commonly employed to study the patterns and distribution of infectious and chronic diseases [11,12], most studies examining geographical differences in risk factors surrounding obesity have been limited to developed countries [13], including characteristics such as high traffic and poverty in Quebec [14], homicide rates and green space in New York City [15], and aspects of the nutrition and physical activity environments in the northwest US [16]. Other studies have correlated risk factors for obesity, such as physical inactivity to neighborhood green space [17] and walkability [18].

In one of the first studies analyzing the geographic distribution of overweight in a developing country, and in particular sub-Saharan Africa, Pawloski *et al.* [19] used longitudinal Demographic and Health Survey data to conclude that overweight and obesity had increased in Kenya from 2003 to 2009, and showed through a Getis-Ord Gi* analysis that overweight clustered in mothers and their children. Another analysis in northeast China found that combined overweight and obesity prevalence in urban children and adolescents was high (33% for males and 18% for females), whereas prevalence was intermediate in wealthy rural areas and still very low in impoverished rural areas [20]. While these studies both identified geographic areas with high obesity, they linked hotspots for overweight with few contributing risk factors, thereby limiting our understanding of the overall implications for designing policies and interventions to encourage healthy weight. We address this gap by using GIS techniques to identify hot spots for underweight and overweight, and correlating geographic data with ordinary least squares (OLS) and multinomial logistic regression of sociodemographic risk factors including age, marital status, education, income, rural/urban residence, and contextualizing this within population-based risk factors including proximity to refugee settlements and cash crop production.

We chose Uganda as a representative low-income country in sub-Saharan Africa because of its diverse geography and ethnic groups, relative political stability, and availability of recent geographic and statistical data from the 2011 Demographic Health Survey. Overweight and obesity, as well as associated chronic diseases such as hypertension [9] and diabetes [21], are quickly becoming more prevalent in Uganda [21,22,23,24]. The national prevalence of overweight and obesity among adult females ages 15–49 is 19%, but areas such as the capital, Kampala, have much higher levels, with 40.4% of women being overweight and 13% being obese [24]. Women are more likely to be overweight than men [21]; a small study in eastern Uganda revealed that overweight was associated with being female, increased age, high socioeconomic status, and peri-urban residence [21]. Even as overweight is increasing, malnutrition persists in Uganda. The prevalence of underweight in children under 5 actually increased from 28% in 2004–2006 to 38% in 2010–2012 [25], and iron deficiency is projected to cause as many as 15,000 maternal deaths by 2015 [26].

In addition, Uganda was also selected for this analysis because the authors are currently conducting pilot studies to identify and target interventions for obesity based on the Analysis Grid for Environments Linked to Obesity (ANGELO) framework developed by Swinburn *et al.* [27]. This framework was developed to focus on microenvironmental settings (e.g., neighborhood food stores) and macroenvironmental sectors (e.g., health care systems) that act as preventive or aggravating factors to obesity risk. The current analysis will help us to better target areas for future studies and the engagement of stakeholders in Uganda. Previous studies analyzing the risk factors for overweight and obesity in Uganda have failed to account for the vast diversity in the agricultural landscape, socioeconomic factors, and political stability [28]. Our statistical and geospatial analyses show that publicly available datasets such as the DHS can be used to assess the burden of underweight and overweight and associated risk factors—both geographically and by sociodemographic characteristics—and thus provide a basis for targeted policies and interventions.

## 2. Experimental Section

### 2.1. Data Source

We utilized publicly available data from the 2011 Uganda Demographic and Health Survey (DHS). These health surveys are nationally representative, population-based surveys that provide indicators of demographics, health, and nutrition [24]. This comprehensive household survey (9,033 households in 404 enumeration areas) included a questionnaire targeted to women. The Woman’s Questionnaire asked eligible women ages 15–49 in each of the households about their background, work, birth history, fertility preferences, breastfeeding, and vaccinations, in addition to other health indicators. The survey was conducted at a multistage cluster level with each cluster comprising 5–10 women per set of geographic coordinates [24]. Each observation includes information about the target female of reproductive age in each household, up to six children, and household characteristics.

The data originally consisted of a sample of 8,674 rural and urban women and 2,336 children under age 5. However, after exclusions, the sample consisted of 2,420 adult females and 1,099 child observations. We excluded observations that lacked height and weight for adults (5,912 cases) and height, weight, age, and sex for children (1,237 cases). We excluded adults with extreme body mass index values (BMI > 80; 54 cases), and pregnant women (288 cases). We also excluded children younger than 6 months of age who were likely breastfeeding, because childhood overweight and obesity generally develop after the introduction of supplemental foods [29]. The majority of infants in Uganda are not exclusively breastfed after 6 months, so we included children ages 6 months to 5 years [30]. GIS coordinate data (latitude and longitude) from the 2011 DHS database were then merged with demographic data for our final sample. Based on a comparison with excluded cases, our final sample was representative both geographically and demographically of the entire sample. All regions across Uganda were represented by approximately 30% of the original DHS sample with BMI data for mothers and children [24].

### 2.2. Anthropometric Measures

Adults were classified as overweight or obese according to the World Health Organization (WHO) definition [3], with a BMI of less than 18.5 kg/m^2^ classified as underweight, 18.5 to 25 kg/m^2^ comprising normal weight, greater than 25 kg/m^2^ overweight, and over 30 kg/m^2^ being obese. Overweight for children was classified according to the 2006 WHO Child Growth Standards as being two standard deviations (+2 SD) above the median weight-for-height based on the child’s specific age and sex [31].

### 2.3. Explanatory Variables

Explanatory variables were chosen based on basic characteristics and established correlations with obesity [19,23,24]. The marital status of adult women was recategorized as *ever married* and *never married*. The *ever married* category included divorced, separated, widowed and currently married adult women. Age was defined as age of women in years at the time of the survey*.* Mothers’ education level was recorded as number of years women spent in school, which ranged from 0 to 18 years of education. It was included in the regression analysis as a continuous variable. The residence of adult women was recorded as *rural* or *urban* based on the Ugandan census [32]. A wealth index constructed using household assets and services data via principal components analysis by the administrators of the DHS was divided into five quintiles: very poor, poor, medium, rich and very rich. For simplicity of analysis we combined very poor and poor together to form the *low wealth* *category*, rich and very rich to form the *high wealth category* and the medium quintile to form the *medium wealth category.* In order to provide context for the wider ecological, economic and political factors that may be impacting underweight and overweight risk, we also created regional measures of political instability and agricultural production. Using data on humanitarian response programs [33] and major cash crop farming [34], we categorized regions as being *conflict prone* based on the presence of refugee camps and settlements, or as *cash crop producing* based on the presence of major coffee, cotton, or tea production.

### 2.4. Statistical Analysis

We used both OLS and multinomial logistic regression to examine demographic and socioeconomic factors that contributed to weight status for mothers and their children. OLS regression has advantages over the categorical estimation methods because it uses all the data points during the estimation. However, it does not provide the probability of moving from normal weight to underweight or from normal weight to overweight or obese as an effect of each risk factor. Therefore, in addition to OLS, we used multinomial logistic regression to estimate the shift in risk of mothers’ weight status due to underlying socioeconomic and demographic risk factors. The regression analysis was performed using Stata-SE version 12.0 (Statacorp, College Station, TX, USA, 2011).

### 2.5. Spatial Analysis

We used ArcGIS version 10.1 (ESRI, Redlands, CA, USA, 2011) to investigate the clustering of underweight and overweight or obesity. Spatial analysis was performed at the cluster level, composed of an average of 5 to 10 aggregated households. The areas with DHS sample clusters did not necessarily overlap with administrative boundaries. Rather, these clusters were GPS coordinate readings taken at the nearest town or community center for an artificially designated area for survey by DHS. Using this point data, we performed Getis-Ord Gi* analyses [35] for local clusters in order to measure global and local clustering of BMI for mothers and to calculate weight-height-age percentiles for children and residual risk factors for mothers and children. Getis-Ord Gi* [35] was an appropriate analytical method for detecting hot spots (and cold spots) for underweight and overweight mothers and children because we have point data based on latitude and longitude coordinates at the center of DHS clusters of sample households.

As previously discussed, the unit of spatial analysis is a cluster of sample households as designated by DHS. In order to prevent the exclusion of local factors by imposing sharp boundaries, the concept of the “zone of indifference” was used for the distance a hot spot boundary covers. Using this method, the neighboring sample households within the specified critical distance boundary of a target DHS sample-household cluster are included in analyses for designating the cluster as a hot spot (or cold spot). Once the specified critical distance boundary is reached, the level of impact of the neighboring clusters is reduced [36]. The computation of *GiZ* score for local Getis-Ord Gi* [35] is given as:

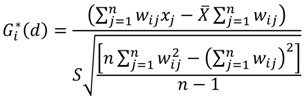
(1)
where 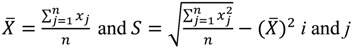 are geographical locations (coordinates) and *x_i_* and *x_j_* are values of the variables under study (BMI for adults and BMI percentile for children) at the coordinates *i* and *j*. The variable *w_i j_* stands for the association weights for coordinates *i* and *j* at distance *d*.

In contrast to measures of global spatial autocorrelation, which return a single-value *z* score for each variable (indicating the degree of regional clustering across the entire distribution of that variable), measures of local spatial autocorrelation produce one value for each location for each variable, indicating the degree to which that particular location is part of a hot spot or cold spot [37].

The local Getis-Ord Gi* statistic compares the local mean rate (the mean of the rates for a cluster of DHS sample households and its nearest neighboring cluster of DHS sample households) to the global mean rate (the mean of the rates for all clusters of DHS sample households). It produces a *z* score and a *p*-value for each cluster of DHS sample households, reflecting whether the differences between the local and global means are statistically significant or not. A statistically significant positive *z* score indicates a hot spot of high rates, meaning that the values are so abnormally high that the chances are very low that the spatial clustering results from random processes. Similarly, a statistically significant negative *z* score for a DHS sample-household cluster indicates local clustering of especially low rates, or a cold spot [35,38]. We used a 95% statistical significance level, which corresponds to *z* scores >1.96 and <−1.96 as cut-off points for significant hot spots and cold spots, respectively.

## 3. Results and Discussion

### 3.1. Risk Analysis

Table 1 summarizes sociodemographic characteristics and weight status of adult women and children in our sample. Out of the 2,420 women, 19% were overweight or obese. Eight percent (*n* = 195) of the women were underweight, 73% (*n* = 1,756) were normal weight, 15% (*n* = 363) were overweight, and 4% (*n* = 106) were obese (data not shown). Urban residents were more likely to be overweight or obese (32%) than rural residents (13%). Overweight was significantly correlated with wealth: only 6% of women in the lowest wealth class were overweight or obese, whereas 30% of women in the wealthiest class were overweight or obese. Education followed the same trend: 13% of women with no education were overweight, whereas overweight increased to 16% with primary level education, to 25% with secondary level education, and to 41% with above secondary level education. Overweight or obese women were also more likely to have overweight children (19%) compared to their normal-weight or underweight counterparts (14%) (data not shown).

**Table 1:** Sociodemographic characteristics and weight status of the sample of adult females (*N* = 2,420) and children (*N* = 1,099). Statistically significant differences were observed for all sociodemographic characteristics between normal/underweight and overweight/obese groups; *An asterisk indicates a statistically significant trend (*p* < 0.001).

Variables	Normal/Underweight	%	Overweight/Obese	%
*Sample population*	*1,951*	*81*	*469*	*19*
*Residence*				
Urban	522	68	248	32
Rural	1,429	87	221	13
Wealth class *				
Low	780	94	53	6
Medium	335	87	51	13
High	836	70	365	30
Education *				
None	291	87	45	13
Primary	1,131	84	217	16
Secondary	435	75	141	25
Above secondary	94	59	66	41
*Children’s BMI percentile category*	*951*	*86*	*148*	*13*

**Table 2:** Ordinary least square regression analysis relating socioeconomic and demographic risk factors for maternal and child weight. Reference categories are *never married* for marital status, *no education* for maternal education level, *low wealth* for wealth class, *non-cash crop producing* *region*, and *non-conflict prone region*. SE = standard error. ** *p* < 0.05, *** *p* < 0.001.

Variables	Coefficients			
	Mothers (*N* = 2,420)	SE	Children (*N* = 1,099)	SE
Maternal BMI			0.015 ***	0.004
Age	0.044 ***	0.010	0.038 **	0.012
Marital status: *ever* *married*	1.002 ***	0.21		
Residence: *urban*	1.079 ***	0.19		
Maternal education level	0.054 ***	0.022	−0.003	0.003
Wealth class: *medium*	0.75 ***	0.24		
Wealth class: *high*	1.61 ***	0.23		
Cash crop producing region: *yes*	0.75	0.18		
Conflict prone region: *yes*	−0.81	0.23		
Intercept	18.61 ***	0.31	−0.003	0.003
R^2^	18.17%		4.52%	

Multinomial logit regression showed that being underweight was significantly and negatively associated with being wealthy or ever married, whereas overweight was significantly and positively associated with increasing age, high wealth status, and residence in an urban area (Table 3). The odds of being overweight increased to 2.94 as a woman’s wealth status increased from low to high (Table 3). Residence in a conflict-prone region was protective (OR = 0.48) against overweight/obesity risk (Table 3).

**Table 3:** Multinomial regression results for the association between socioeconomic and demographic risk factors and weight status of adult women in the sample (*N* = 2,420). References for the risk factors are *never married* for marital status, *rural* for residence, *no education* for education level, and *low wealth* for wealth class, *non-cash crop producing region*, and *non-conflict prone region*. The odds ratios use normal weight status as the reference. Normal weight is the denominator. SE = standard error; CI = confidence interval. * *p* < 0.1, ** *p* < 0.05, *** *p* < 0.001.

Variables	Underweight		Overweight and obese	
	Log odds ratio (SE)	Odds ratio [95% CI]	Log odds ratio (SE)	Odds ratio [95% CI]
**Age**	0.022 (0.011)	1.022 [1.000,1.045]	0.044 *** (0.0075)	1.045 *** [1.030,1.052]
**Marital status: *evermarried***	−0.66 ** (0.25)	0.512 ** [0.32, 0.84]	0.36 * (0.17)	1.43 * [1.034, 1.98]
**Residence: *urban***	0.0029 (0.27)	1.003 [0.59,1.69]	0.56 *** (0.14)	1.76 *** [1.34, 2.29]
**Education level**	−0.028 (0.028)	0.97 [0.92, 1.027]	0.031 * (0.016)	1.032 * [1.001, 1.063]
**Wealth class: *medium***	−0.89 ** (0.27)	0.41 ** [0.24, 0.69]	0.34 (0.23)	1.41 [0.89, 2.19]
**Wealth class: *high***	−0.92 *** (0.26)	0.39 *** [0.24,0.66]	1.079 *** (0.19)	2.94 *** [1.99,4.35]
**Cash crop producing region: yes**	−0.32 (0.22)	0.72 [0.47, 1.11]	0.23 (0.13)	1.26 [0.97, 1.62]
**Conflict prone region: yes**	0.17 (0.22)	1.19 [0.78, 1.81]	−0.73 ** (0.24)	0.48 ** [0.29, 0.78]
**Intercept**	−1.72 *** (0.35)		−3.98 *** (0.28)	

### 3.2. Spatial Analysis Results

**Figure 1:**
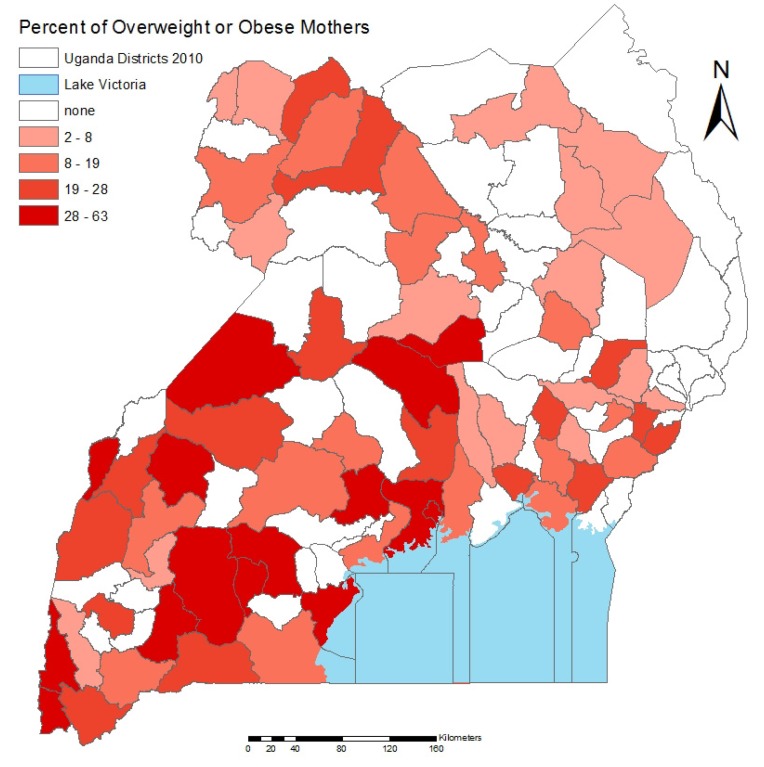
The percentage of overweight mothers in Uganda shown as quintiles, by district, where darker districts experience higher levels of overweight and obesity.

Figure 1 displays the percentage of overweight and obese mothers in Uganda by district, ranging from 0% to almost 64% overweight in some regions. Regions in the Southwest are considerably overrepresented here, with the districts including Kampala, the capital, and Mbarara, the third largest city in Uganda, having two of the highest rates of overweight and obesity, ranging from 19% to 63%.

**Figure 2:**
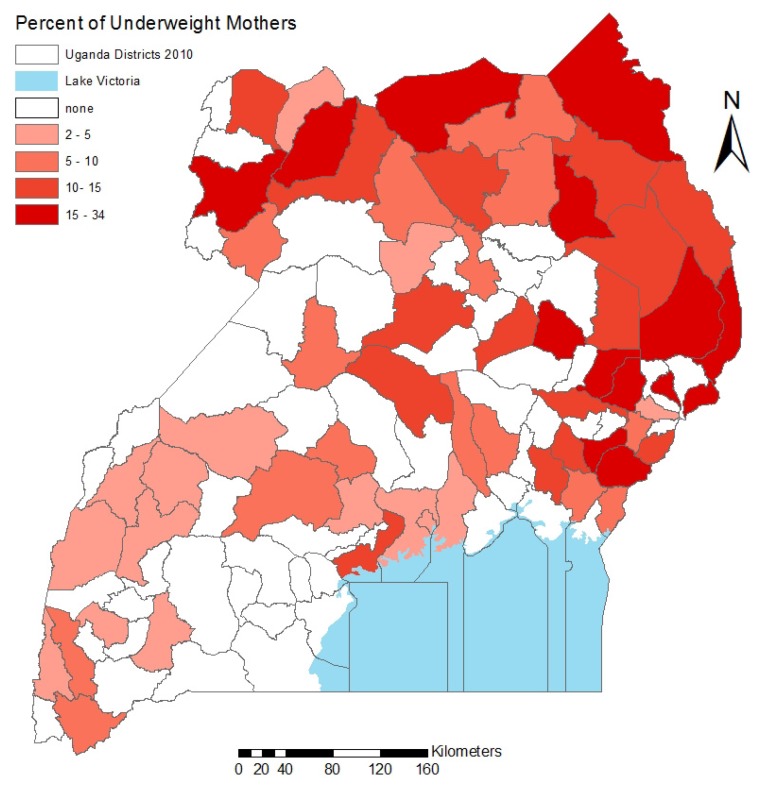
The percentage of underweight mothers in Uganda as quintiles, by district, where darker districts experience higher levels of underweight.

Figure 2 shows the percentage of underweight mothers by district, ranging from 0% to 34%. Not surprisingly, the distribution is almost completely the reverse of the overweight map. The Karamoja region in the northeast has an extremely high prevalence of underweight (15–34% region-wide), and the east central region is also over-represented.

**Figure 3:**
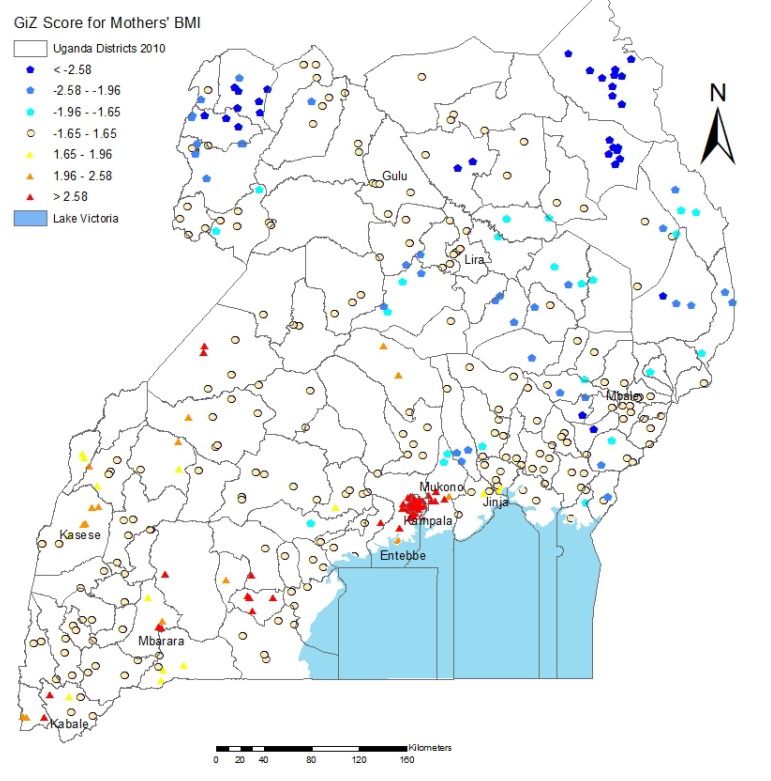
Hot and cold spots of maternal BMI. Dark red dots represent hot spots of high maternal BMI, and dark blue dots represent cold spots of low maternal BMI at the 95% statistical significance level. Each dot represents a cluster of DHS sample households, while the boundaries shown are districts. Dots inside Lake Victoria are located on islands.

Figure 3 shows the Getis-Ord Gi* Hot Spot analysis for overweight and obese women in Uganda. This shows a striking trend, with the entire southern region facing a significant burden of overweight and obesity among adult women, while the northern regions suffer from underweight. All but one of the regions with the highest clustering of overweight are in a major city; these are the major cities of Kampala, Mbarara, Masaka and a smaller city, Kabale [32].

**Figure 4:**
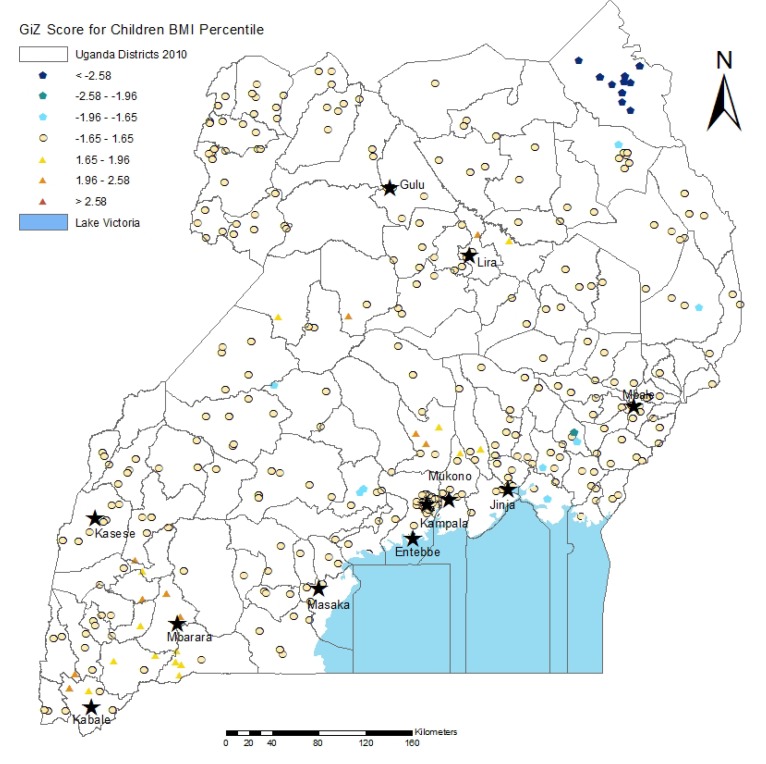
Hot and cold spots of child BMI percentile. Dark red dots represent hot spots of high child BMI percentile, and dark blue dots represent cold spots of low child BMI percentile at the 95% statistical significance level. Each dot represents a cluster of DHS sample households, while the boundaries shown are districts. Dots inside Lake Victoria are located on islands.

As noted in the experimental section, BMI for children is calculated differently than BMI for adults, with children at or above the 85th percentile for weight, height, and age being considered overweight. The Getis-Ord Gi* Hot Spot analysis of clusters in children (Figure 4) shows that the prevalence of both overweight (16.70%) and underweight (6%) is much lower in children than in their mothers, but that many of the hot spots for overweight children are found in the regions where the greatest amount of maternal overweight is observed. Again, overweight is clustered in the Kampala and Southwest districts.

### 3.3. Discussion

Our results suggest that there is a significant burden of overweight in Uganda in urban areas and in wealthier regions of the country. Overweight and obesity are correlated with higher age, living in an urban area, and high wealth. The Southwest region and the capital city of Kampala had the highest rates of obesity, with the rate of overweight reaching 40%. The prevalence of overweight in children was much lower overall (17%), but is still significant in Kampala, several major cities in the southwestern corner, and two major northern cities. Overweight children were more likely than their normal-weight or underweight counterparts to have overweight or obese mothers. However, in the area surrounding the major northern city of Lira, two significant hot spots emerged for children even though no such significant hot spots occurred for their mothers. This validates the results of Pawloski *et al.* in Kenya, in which most hot spots emerged for both mothers and children, even though some hot spots for overweight in children occurred with normal-weight mothers, and some hot spots for mothers occurred with normal-weight children [19].

The prevalence of overweight and obesity was decidedly lower in the North. Even though overweight was associated with urban residence, the three largest cities in the North (Gulu, Lira, and Mbale) did not emerge as hot spots for overweight and obesity in mothers or children. This is likely related to the high levels of displacement and poverty accompanying the 21-year civil war in the North between the Ugandan government and rebel groups, including the Lord’s Resistance Army [39,40,41], which is borne out in our analysis. The Karamoja region in the far Northeast is one of the most malnourished in the country, and the North suffers from the highest level of food insecurity [28], possibly explaining some of the variability within the country.

While the issues affecting obesity risk are complex, the Southwest likely has a high prevalence of overweight because of its relative political stability, greater presence of tourism and nongovernmental organizations that provide economic support and supplemental nutrition, increased economic opportunities, higher rainfall and cash crop yields, and subsequent higher level of food security. Furthermore, these factors have led to better education and higher wealth, which also correlate with higher weight status in Uganda [24,28].

### 3.4. Strengths and Limitations

Our study provides insights into the demographic and geographic correlates of weight status in Uganda. Despite the limitations associated with utilizing the DHS for our analysis (described below), this survey remains the largest nationally representative dataset including weight status coupled with geographic coordinates for Uganda. Moreover, van Wesenbeeck, Keyzer, and Nube have validated the use of the DHS to survey nutrition-related health outcomes in Africa [42].

Our study has several limitations. First, a substantial subset of adult women from our initial sample was excluded due to the lack of anthropometric measurements necessary for calculating BMI. Exclusions notwithstanding, after comparing our final sample with the excluded cases, we found that our final sample was representative both geographically and demographically of the entire sample. Second, the DHS does not include items on health behaviors that are related to obesity risk, such as daily physical activity levels, which makes it challenging to draw inferences about other individual-level factors that may lead to overweight or obesity. Third, aside from young children and females of child-bearing age, specific population groups are not well represented, although the 2011 DHS did survey adult males. Fourth, the DHS aggregates groups of 5–10 women into single sets of coordinates, thereby limiting the exact point data. However, since this was a countrywide study with small cluster areas that have very similar environmental characteristics, this point-data limitation should not affect the conclusions drawn from our study.

## 4. Conclusions

Our study is one of the first spatial analyses of overweight and obesity in Uganda. It is currently one of the few and most in-depth focused on a low-income country, accounting for both individual- and broader socio-political factors age such as age and internal displacement that may influence population-based differences in weight status. We used GIS techniques to show regional variation in maternal-child overweight and obesity in Uganda. Our analysis illustrates how geospatial and statistical tools can be used with publicly available datasets—such as the DHS—to elucidate some of the risk factors and sociodemographic characteristics related to overweight and obesity. In so doing, we show that the DHS can be better utilized as a tool for tracking the long-term implications of undernutrition and overnutrition in Uganda and sub-Saharan Africa.

Consequently, our work underscores the growing value of using geostatistical tools to elucidate the etiology of overweight and obesity and the increasing rates of chronic disease in sub-Saharan Africa. Future research should focus on how regionally specific environmental factors may affect the risk of overweight and obesity. Collection of more in-depth data concerning other risk factors for chronic disease, such as physical activity levels, is necessary for a comprehensive understanding of the development and pathology of obesity in sub-Saharan Africa. The DHS and other nationally representative surveys that provide spatial coordinates can be utilized for determining spatial clusters and correlates for overweight and obesity that may not be obvious using traditional regression methods, and can provide a starting point for developing tailored interventions and policies to address the impact of noncommunicable diseases in Uganda and sub-Saharan Africa.
